# Capillary isoelectric focusing after sample enrichment with immunoaffinity chromatography in a single capillary

**DOI:** 10.1038/srep39221

**Published:** 2016-12-15

**Authors:** Kiyohito Shimura, Toshihiko Nagai

**Affiliations:** 1 Division of Advanced Chemistry, Fukushima Medical University, 1 Hikarigaoka, Fukushima, 960-1295, Japan

## Abstract

For accurate micro-scale quantification of a specific protein in biological fluids, immunoaffinity chromatography (IAC) and isoelectric focusing (IEF) were combined in a single fused-silica capillary. The inner wall of the capillary was coated with an anti-E-tag antibody at the inlet side to form an IAC column, and polydimethylacrylamide, a neutral polymer, at the outlet side to form the capillary for IEF. After loading a sample, the whole capillary was filled with a carrier ampholyte solution. An anode solution, an acid, was then introduced to fill only the IAC column segment. Focusing was started with a pressure that balances with the electroosmotic flow produced in the acidified IAC column. Fluorescence-labeled recombinant Fab with an E-tag spiked at 16 pM to 10 nM in 50% serum was separated and detected with high precision. The coupling principle allows rapid and high-resolution IEF analysis of a protein in a biological sample without any loss of the immunoaffinity captured protein.

The combination of electrophoretic separation and immunochemical detection, such as immunoelectrophoresis^1^ and the currently-used western blotting^2^, has provided strong evidence for the presence of a specific protein in a biological sample. The need for a combination is because immunochemical detection alone could be affected by false signals, and electrophoretic separation alone is not enough to detect a protein that is present at very low concentration in complex biological samples. Immunoaffinity capillary electrophoresis (IACE) was developed as a combination of immunoaffinity and electrophoresis on a micro scale^3^,^4^,^5^. So far, zone electrophoresis has been successfully used for separation in IACE^6^,^7^. Although isoelectric focusing (IEF) provides a higher resolution than zone electrophoresis for the separation of proteins^8^,^9^,^10^,^11^,^12^, to our knowledge a successful coupling of immunoaffinity chromatography (IAC) and capillary isoelectric focusing (CIEF) has yet to be reported.

We have recently succeeded in coupling nickel-chelate affinity chromatography and CIEF in a single capillary, which we call the unified capillary^13^. The inner wall of the capillary is coated with an iminodiacetate-derivative, poly(3-*N,N*-dicarboxymethylamino-2-hydroxypropyl methacrylate), at the inlet side and polydimethylacrylamide (PDMA) at the outlet side. The former coating holds nickel ions that act as an affinity ligand for hexahistidine (6xHis)-tagged proteins, and the latter coating suppresses electroosmotic flow (EOF) in a fused-silica capillary, providing a suitable environment for IEF. To achieve success, the nickel-chelate column segment must be filled with an anode solution before starting IEF separation. Otherwise, a high EOF would be produced in the nickel-chelate column, and its direction would change from anode-to-cathode to cathode-to-anode. This change of EOF direction is caused by the pH change in the affinity column during the focusing, *i.e.*, the process involving establishment of a pH gradient. An anode solution such as 100 mM phosphoric acid has a considerably higher electric conductivity than a carrier ampholyte solution. This means that the voltage drop in the capillary segment filled with an anode solution is considerably smaller than that in the segment filled with a carrier ampholyte solution. Filling the affinity column with an anode solution can thus effectively reduce the voltage gradient in this segment and the EOF accordingly. The acidification with the anode solution can also prevent the reversion of the EOF direction. The residual small EOF can be managed by applying overwhelming pressure at the anodic end. This configuration provides an ideal condition for IEF in the neutral polymer-coated capillary segment, *i.e.*, a very low level of EOF and a very low hydrodynamic flow. On the other hand, a turbulent flow should be produced in the affinity column segment due to the counteracting EOF and pressure-driven flow, but this turmoil does not extend to the neutral polymer coated capillary (Fig. 1).

The merits of this unified separation system are: (1) a sample volume larger than the capillary volume can be loaded; (2) salts and unrelated highly abundant proteins that may compromise IEF separation can be removed; (3) the whole proteins that were captured in the affinity column can be analyzed by CIEF without loss; and (4) CIEF is performed under optimal conditions to achieve high resolution separation without any compromise.

In the present report, we applied the unification principle of affinity chromatography and CIEF to the combination of IAC and CIEF. The unified IAC-CIEF provides a fast, easy and reliable method to detect and quantify a specific protein in complex biological fluids at a micro scale.

## Methods

### Chemicals and Materials

The following were obtained from commercial sources: fused-silica capillaries (Polymicro Technologies, Phoenix, Arizona, USA); rubber septa for gas chromatography (Shimadzu type, GL Sciences Inc., Tokyo, Japan); Pharmalyte 3–10 (GE Healthcare Japan, Tokyo, Japan); 3-methacryloxypropyltrimethoxysilane (Shin-Etsu Chemical, Tokyo, Japan); streptavidin, tris(hydroxymethyl)aminomethane (Tris), *N,N*-dimethylacrylamide, *N,N,N′,N′*-tetramethylethylenediamine (TEMED), and 3-aminopropyltrimethoxysilane (Wako Pure Chemical Industries, Osaka, Japan); *N,N′*-disuccinimidyl carbonate (Nacalai Tesque, Inc., Kyoto, Japan); tetramethylrhodamine-5-iodoacetamide dihydroiodide (5-TMRIA, T6006), and 5-carboxymethylrhodamine succinimidyl ester (5-TAMRA, SE; C2211) (Life Technologies Japan, Tokyo, Japan); diisopropylethylamine (Tokyo Chemical Industry, Tokyo, Japan); goat anti-E tag antibody (affinity purified, biotin conjugate) (A190-132B, Bethyl Laboratories, Inc., Montgomery, Texas, USA); and Anti-His-tag-Biotin mouse monoclonal antibody (Code No. D291-6, Medical & Biological Laboratories Co., Ltd., Nagoya, Japan). Fluorescence-labeled peptide isoelectric point (pI) markers were prepared as described previously^14^. Anti-α_1_-antitrypsin recombinant antibody fragment (rFab) and anti-insulin rFab, both of which have an E-tag as a peptide affinity tag at the carboxyl terminal of the light chain, were prepared and labeled with tetramethylrhodamine-5-iodoacetamide at the carboxyl terminal of the Fd chain (VH + CH1 domains), and purified as previously reported^15^,^16^. Anti-insulin rFab with a 6xHis tag was prepared, labeled, and purified as previously described^13^.

### Preparation of unified capillary

Fused silica capillaries (50 μm internal diameter [i.d.], 365 μm outer diameter [o.d.], 60 cm in length) were marked at 25 cm from the end with a felt-tip marker. The shorter side was used as the inlet side. The capillaries were rinsed with 50 μL of acetone using a micro syringe, connected with a Teflon tube, filled with 1 M NaOH, and left for 30 min at room temperature. Next, the capillaries were each rinsed with 50 μL of 0.1 M HCl, water, and acetone, and were dried by sucking air using a micro syringe. Aminosilane solution (5% [v/v] 3-aminopropyltrimethoxysilane, 5% [v/v] water, 90% [v/v] ethanol, to which water was added 5 min before use) was introduced into the capillaries from the inlet side until the meniscus reached the mark^13^,^17^. Both ends were closed by piercing them into a rubber septum, and the capillaries were left to react at room temperature for 20 min. The capillaries were then rinsed with 50 μL of ethanol by injecting the solvent from the outlet, rinsed with acetone, and air-dried by suction. Due to the fast reaction of the aminosilane, the rinse solvents should be introduced from the outlet side, the untreated side, in order to keep the untreated silica surface intact without contact with the aminosilane.

Methacrylsilane solution (10% [v/v] 3-methacryloxypropyltrimethoxysilane, 45% [v/v] acetic acid, 45% [v/v] acetone) was introduced into the capillaries from the outlet side until the meniscus reached the mark. Both ends were closed and left to react at room temperature overnight. The capillaries were rinsed with water and acetone by introducing the solvents from the inlet side, and then air-dried. The PDMA coating was applied to the methacrylate-coated silica surface from the mark to the outlet end as described previously^13^ to form the Amino/PDMA capillaries.

The amino group of the capillaries was activated with *N,N′*-disuccinimidyl carbonate (DSC)^17^. The DSC-reaction mix (DSC 15 mg, diisopropylethylamine 50 μL, and acetone 1.45 mL) was introduced into the Amino/PDMA capillaries from the inlet side, *i.e.*, the amino-functionalized side, until the meniscus reached the marked point. Both ends were closed and left to react at room temperature for 2 h. The capillaries were then rinsed with acetone from the outlet and air-dried. Streptavidin dissolved at 1 mg/mL in phosphate buffered saline (PBS) (2 mM KH_2_PO_4_, 10 mM Na_2_HPO_4_, 2.7 mM KCl, 137 mM NaCl, pH 7.4) was introduced into the activated Amino/PDMA capillaries from the inlet side until the meniscus reached the marked point. Both ends were closed and left to react at room temperature overnight. To inactivate any residual activated amino groups, 20 μL of 0.5 M Tris-HCl, pH 8.0 were injected into the capillaries, which were left at room temperature for 1 h to form Streptavidin/PDMA capillaries. The capillaries were finally rinsed with 50 μL of PBS containing 0.02% NaN_3_. Both ends were connected with a Teflon tube (0.33 mm i.d.) filled with the PBS-NaN_3_, and stored in a refrigerator. Five to ten capillaries were prepared in a single batch of preparation.

### IAC-CIEF

The operation of the unified capillary was carried out using an automated capillary electrophoresis instrument (Beckman-Coulter P/ACE MDQ, Brea, California, USA) with fluorescence detection through a band pass filter (FF01-593/40, Semrock Inc., Rochester, New York, USA), using a 532 nm laser (Model CL532-010, CrystaLaser LC, Reno, Nevada, USA) for excitation. The unified capillary was installed in the capillary cartridge of the instrument with a coolant tube for the 50 cm capillary. When the coolant tube was used, the actual length between the inlet end and the detection point was 38.5 cm, and the distance between the detection point and the outlet end was 10 cm. This made the length of the streptavidin-coated segment 18.5 cm at the inlet side and the length of the PDMA segment 30 cm at the outlet side. The capillary was equilibrated by injecting PBS containing 0.1% Tween 20 (PBS-Tw) at 3.2 psi (24 cm/min linear velocity) for 1 min, and was loaded with 10 ng/μL of biotinylated anti-E tag antibody in PBS-Tw containing 4% glycerol at 0.8 psi (6.0 cm/min linear velocity) for 1 h. The pressure-linear velocity relationship, which fitted well with Hagen-Poiseuille equation, was determined as previously described^13^. The capillary was rinsed with PBS-Tw at 3.2 psi for 5 min to form the anti-E tag antibody/PDMA unified capillary.

**Figure 1:**
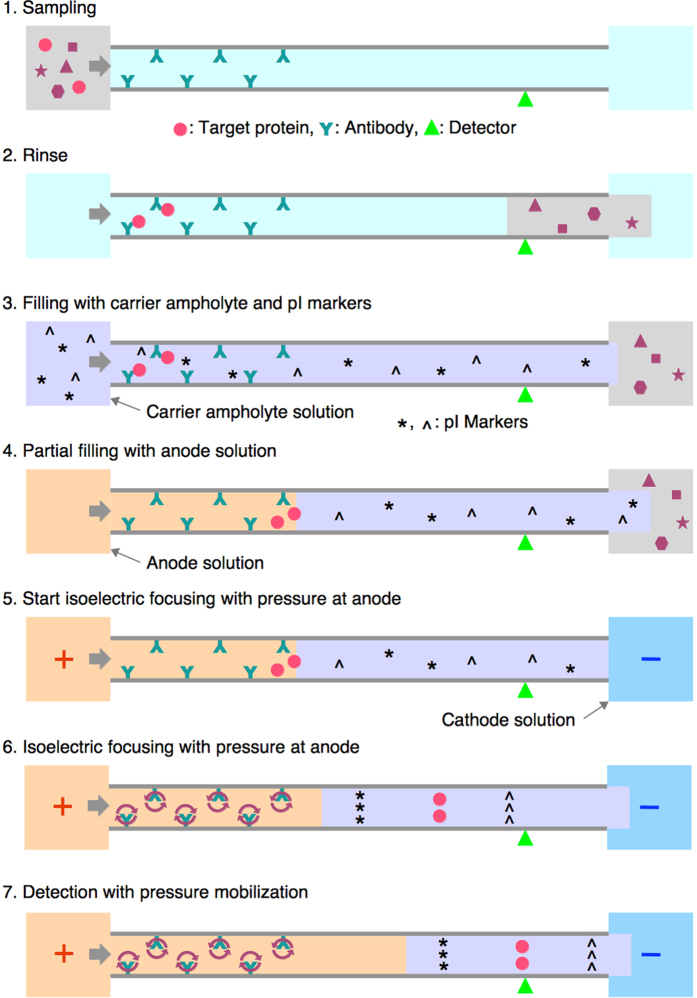
Step-by-step processes of direct coupling of immunoaffinity chromatography and capillary isoelectric focusing. The circular arrows in the immunoaffinity column at steps 6–7 represent local mixing flow in the capillary produced by the counteracting EOF and pressure-driven flow.

To start the analysis, the capillary was washed with PBS-Tw at 50 psi for 1 min. A sample solution containing rFab, which was E-tagged and labeled with tetramethylrhodamine, in 0.1 M Tris-HCl buffer (pH 8.0) containing 0.1% Tween 20 or in PBS-Tw was injected into the capillary at 0.8 psi for 4 min, which corresponds to a sample plug length of 24 cm and to a volume of 0.47 μL (Fig. 1). The capillary was rinsed with PBS-Tw containing 0.5 M NaCl at 3.2 psi for 5 min, and further rinsed twice with 20 mM Tris-HCl (pH 7.4) containing 0.1% (w/v) Tween 20 at 3.2 psi for 0.5 min each from different buffer vials to remove excess salts in the unified capillary and to reduce carryover of NaCl into a carrier ampholyte solution. The carrier ampholyte solution (2.5% [v/v] Pharmalyte 3–10, 0.1% [v/v] acetic acid, 0.6% [v/v] TEMED, 0.1% [w/v] Tween 20), containing 2.5 nM each of the fluorescence-labeled peptide pI markers pI 3.64 and pI 9.56^14^, was injected at 3.2 psi for 2 min, corresponding to a plug length of 48 cm. The anode solution (100 mM phosphoric acid) was then injected at 2.6 psi for 1.0 min, which corresponded to a plug length of 19.5 cm, to fill the immunoaffinity column segment. Focusing was started at a voltage of +25 kV with a pressure of 0.2 psi at the anode, and focusing and mobilization were continued for 30 min. Finally, the capillary was rinsed with PBS-Tw at 50 psi for 1 min.

## Results and Discussion

### Immunoaffinity chromatography on the unified capillary

The chromatographic character of the unified capillary was examined using the anti-E tag antibody-immobilized unified capillary. Fluorescence-labeled rFab containing an E tag (GAPVPYPDPLEPR) was used as a model sample. In our previous report on the coupling of metal-chelate affinity chromatography and CIEF^13^, a low flow rate of about 6 cm/min linear velocity was necessary to achieve an almost full capacity of the affinity column. This is because of the slow mass transfer in the hollow capillary column to achieve a quasi-binding equilibrium between the mobile phase and the immobile phase carrying the anti-E tag antibody on the inner wall of the capillary.

**Figure 2:**
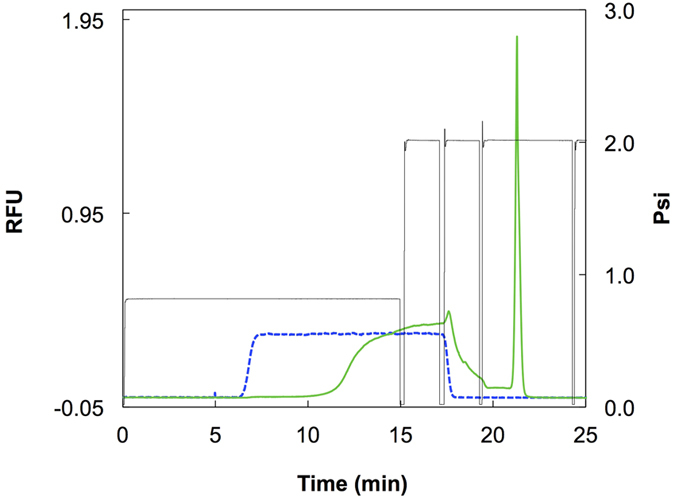
Affinity chromatography of fluorescence-labeled rFab containing E tag on the unified capillary. The unified capillary (50 μm i.d., 48.5 cm in length) was equilibrated with PBS-Tw at 2.0 psi (a linear flow rate of 15 cm/min) for 5 min. The fluorescence-labeled rFab at 100 nM in PBS-Tw was applied at 0.8 psi (6.0 cm/min) for 15 min (1.75 μL, 175 fmol of rFab) on the capillary bearing anti-E tag antibody. The column was washed at 2.0 psi for 2 min each with PBS-Tw containing 0.5 M NaCl, and with 20 mM Tris-HCl (pH 7.4) containing 0.1% (w/v) Tween 20. The bound material was eluted with 100 mM phosphoric acid at 2.0 psi for 5 min (green solid line). Exactly the same procedure was carried out on the capillary before immobilizing the anti-E tag antibody, *i.e.*, only with the immobilized streptavidin (blue dotted line). The pressure (psi) was recorded with the black line. RFU, relative fluorescence unit.

Before testing the antibody column, the column without any antibody was tested as a control experiment using the streptavidin-immobilized unified capillary. When rFab, which is E-tagged and labeled with tetramethylrhodamine, at 100 nM was applied on the streptavidin-immobilized column at a linear flow rate of 6.0 cm/min for 15 min, which corresponds to an imaginary sample plug-length of 90 cm and a volume of 1.8 μL, a plateau of the fluorescence signal was observed, and no fluorescence material was eluted by subsequent irrigation with 100 mM phosphoric acid (Fig. 2, blue broken line). After immobilization of the anti-E tag antibody, the appearance of an elution front was considerably delayed in comparison with that observed for the streptavidin column (Fig. 2, green solid line). Based on the delay of the elution front, the capacity of the column was estimated at 136 fmol/18.5 cm capillary (10 capillaries, the coefficient of variation [CV]=13.5%). The value of the capacity was determined at the first experiment with each capillary to rule out inactivation of the immobilized antibody by the eluent, 100 mM phosphoric acid (see below). Irrigation with 100 mM phosphoric acid eluted bound rFab from the column. Please note that this chromatographic process is adequately described with the theory of frontal affinity chromatography^18^. The product of the concentration of a sample solution and the delay volume of the elution front represents the capacity of the column. The important point is that complete capture of the target molecule in a sample is achieved until the capacity is reached. This makes a striking contrast to batch-wise immunocapture methods, where free target molecules always remain at an equilibrium concentration and thus, escape the capture and are lost.

The rFab containing a 6xHis tag instead of an E tag was tested in the same manner on the anti-E tag antibody column. No adsorption was observed as was the case for the combination of rFab containing an E-tag and streptavidin column. On the other hand, with the combination of rFab containing a 6xHis tag and anti-6xHis tag antibody column, almost the same chromatographic results were obtained as with the combination of rFab containing an E-tag and anti-E tag antibody column. These results indicate that the affinity between the immobilized anti-peptide tag antibody and the peptide tag on the rFab plays a crucial role in the retention of the column.

Reproducibility of the chromatographic run was tested with six cycles of application and elution of 12 fmol of labeled rFab containing an E tag on the same anti-E tag antibody column. The average eluted peak area and the CV were 8.91 × 10^5^ area unit, which is a proportional value to RFU-time integral, and 1.7%, respectively. Quite remarkably, almost identical chromatographic results were obtained even when rFab spiked in 50% serum was used as a sample with an average peak area and CV value of 8.54 × 10^5^ area unit and 2.6%, respectively. This area decrease may partly be explained by the decrease in sample volume due to the increase in viscosity of the sample containing serum at 50%.

To check whether the amount of loaded anti-E tag antibody was enough to saturate the immobilized streptavidin, the capacity of the anti-E tag column was estimated as a function of the loading time of the biotinylated anti-E tag antibody, being based on the delay of the elution front of 100 nM rFab containing E-tag, as described above. The results were as follows for the loading time and the capacity: 10 min, 25 fmol; 30 min, 82 fmol; 60 min, 129 fmol; 120 min, 148 fmol; and 300 min, 139 fmol. The plateau level was almost reached at 60 min, which is equivalent to the loading input of approximately 400 fmol of IgG, and was used in the following experiments.

### Optimization of pressure setting

In a previous investigation of the coupling of metal-chelate affinity chromatography and CIEF, we found that successful separation was achieved when CIEF was started after immersing the affinity column segment in the anode solution, which suppresses the EOF produced in the affinity column and fixes the direction of EOF^13^ (Fig. 1). The residual anodic EOF was balanced with pressure applied at the anodic end. To find the optimum pressure setting for balancing EOF and mobilizing the pH gradient through the detection point, “Zebra” experiments were carried out in the same way as those performed for the optimization in coupling metal-chelate affinity and CIEF^13^.

**Figure 3:**
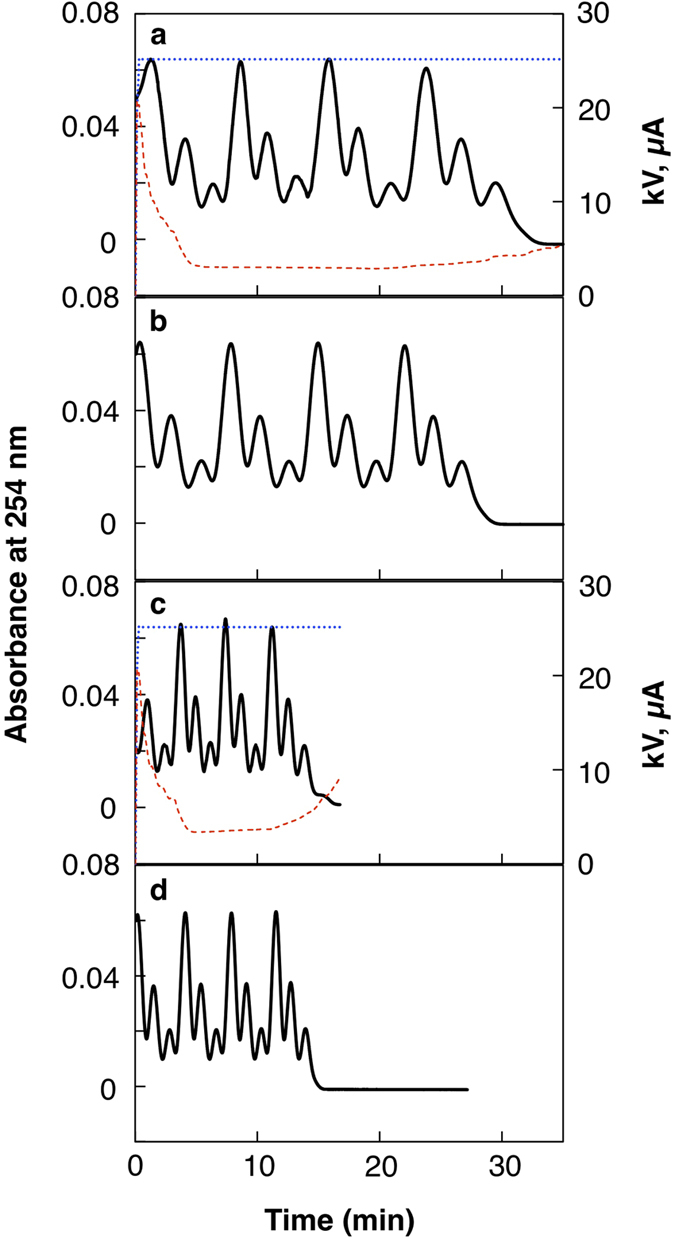
Zebra experiments for optimizing pressure setting. The unified capillary, which consisted of anti-E tag antibody capillary column for 18.5 cm and PDMA-coated capillary for 30 cm, was installed in a capillary electrophoresis instrument with the antibody column side at the anode and the inlet side. A detection window was set at 10 cm from the outlet end for UV absorption detection at 254 nm. Acrylamide as a neutral marker at 40, 20, and 10 mg/mL in the carrier ampholyte solution was consecutively injected from the inlet (anodic side) at 0.5 psi for 10 s, between each of which the carrier ampholyte solution alone was injected at 0.5 psi for 20 s. In total, 10 sets of the triplets were injected to fill the 48.5 cm capillary with the Zebra pattern of acrylamide. Then, an anode solution, 100 mM phosphoric acid, was injected at 2.6 psi for 1.0 min (a plug length of 19.5 cm) to fill the immunoaffinity column segment of the capillary. IEF was started with a voltage of + 25 kV and with a pressure of 0.1 psi (**a**) or 0.2 psi (**c**) at the anodic end. Control experiments were performed without voltage application at 0.1 psi (**b**) and 0.2 psi (**d**). Solid lines, UV traces detecting acrylamide; blue dotted lines, voltage; red broken lines, current.

Acrylamide at three different concentrations in the carrier ampholyte solution was consecutively injected from the inlet (anodic side), between each of which the carrier ampholyte solution alone was injected, to form a triplet Zebra pattern of acrylamide in the capillary. The anode solution, 100 mM phosphoric acid, was then injected to fill the immunoaffinity column segment of the capillary. IEF was started with a pressure of 0.1 psi or 0.2 psi at the anodic end. The Zebra pattern of acrylamide, a neutral molecule, was detected with ultra-violet absorption to observe bulk fluid flow inside the capillary (Fig. 3).

With a pressure of 0.1 psi, a small delay of mobilization was observed due to the residual anodic EOF (Fig. 3a) in comparison with a control mobilization without voltage (Fig. 3b). The delay was more apparent in the early part of focusing, where the first peak broadened, and the peak width soon became almost identical to those of the control mobilization without voltage. This rapid reduction in the electroosmosis toward the anode in the early part of the focusing process is ascribed to the rapid decrease in the conductivity of the carrier ampholyte solution during this period as it is represented in the rapid drop of current (Fig. 3a red line, 0–4 min). The decrease in the conductivity of the carrier ampholyte solution should increase the voltage drop in the carrier ampholyte solution-filled segment, and cause a decrease in the voltage drop in the anode solution-filled segment under the constant voltage setting, resulting in reduction of EOF in the IAC column^13^. With a pressure of 0.2 psi, the difference of the mobilization pattern between the IEF condition (Fig. 3c) and pushing pressure alone (Fig. 3d) was very subtle. The difference in the start point of the detected Zebra pattern in the different experiments was ascribed to the accumulated error of the repeated injection process, and was not an issue in the experiments.

The results of the Zebra experiment indicate that the immersion of the immunoaffinity column segment with the anode solution suppressed its EOF very efficiently. Both pressure settings should allow the detection of the pH gradient formed in the unified capillary. The smallest pressure setting allowed in this instrument was 0.1 psi, and the second smallest pressure was 0.2 psi. The latter pressure was chosen for the following experiments taking into consideration shorter analysis time. This pressure setting allows the detection of the whole pH gradient within 15–20 min after start of IEF. In real use of the unified capillary, however, we experienced elongation of the detection time of the whole pH gradient of up to 25 min at 0.2 psi. In a simple CIEF separation in a capillary coated with PDMA only, a pressure setting of 0 psi for 7 min and 0.2 psi thereafter is appropriate for the focusing and detection of the whole pH gradient within 30–35 min. Thus, the difference in pressure settings between a simple CIEF analysis and an IAC-CIEF analysis under these conditions is small, but continuous application of pressure at the anode is necessary to keep the immunoaffinity column covered with the anode solution. It was also confirmed that there was no obvious change in the EOF in the IAC column after prolonged exposure to the anode solution, *e.g.*, 100 mM phosphoric acid for 16 h, the conditions which considerably reduced the capacity of the immunoaffinity column (see below).

### Coupling of IAC and CIEF in the unified capillary

The rFab containing E tag was first adsorbed on the anti-E tag antibody column, and was then eluted and separated by CIEF in the unified capillary (Fig. 1). After sample application, the solution in the unified capillary was replaced with the carrier ampholyte solution containing two fluorescence pI markers, followed by the anode solution, 100 mM phosphoric acid, to fill the segment of the immunoaffinity column. The anode solution is also very effective for the elution of bound proteins from the immunoaffinity column.

**Figure 4:**
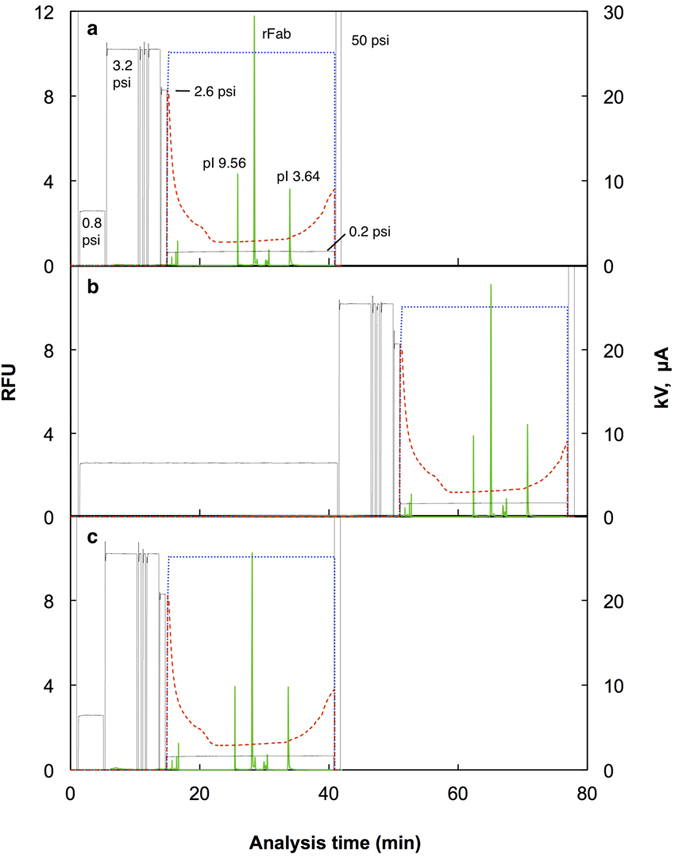
Analysis of rFab using the unified capillary. The rFab was applied to the unified capillary consisting of anti-E tag antibody column (50 μm i.d.; 18.5 cm long) and PDMA-coated capillary (50 μm i.d.; 30 cm long). After sample application at 0.8 psi, the capillary was rinsed and filled with the carrier ampholyte solution containing 2.5 nM each of pI 9.56 and 3.64 markers at 3.2 psi. The anode solution, 100 mM phosphoric acid, was then injected to fill the antibody column segment of the capillary at 2.6 psi for 1 min and IEF was started by applying +25 kV and 0.2 psi at the antibody column side, and with 100 mM NaOH as the cathode solution. The focused protein and markers were detected with a fluorescence detector placed at 10 cm from the cathodic end. (**a**) 50 nM of labeled rFab in PBS-Tw, 4 min injection, 24 fmol; (**b**) 5 nM of labeled rFab in PBS-Tw, 40 min injection, 24 fmol; (**c**) 50 nM of labeled rFab in 50% serum, 4 min injection, 24 fmol. Green solid lines, fluorescence; blue dotted lines, voltage; red broken lines, current; black lines, pressure.

Focusing was started with a pressure setting as it is described in the previous section both to counteract the residual EOF and to slowly mobilize the pH gradient to the detection point. The peaks of the rFab and two pI markers were identified for 50 nM rFab sample in PBS-Tw applied at 0.8 psi for 4 min (0.47 μL, 24 fmol) (Fig. 4a). The pI of the rFab was calculated to be 7.69 ± 0.04 (standard deviation) from this experiment (n = 8), assuming a linear relationship exists between the detection time and the pI values of the two pI markers, *i.e.*, pI 3.64 ± 0.02 and 9.56 ± 0.03. The pI value of rFab was previously determined to be 7.6 using the pI markers having closer pI values to that of the rFab^16^. The error in the pI estimation of 0.09 in this experiment can be ascribed to the use of two pI markers that are distant from the pI value of the rFab, since a non-linear relationship between pI value and detection time is common in the pH gradients formed with carrier ampholytes^19^. A ten times diluted sample was applied for a period ten times longer than the above experiment, *i.e.*, 5 nM rFab in PBS-Tw at 0.8 psi for 40 min (4.7 μL, 24 fmol), and the trapped rFab was separated by CIEF (Fig. 4b). An almost identical separation pattern to that of the one-tenth sampling time for the ten-times higher concentration was obtained. The pI value of the rFab was calculated to be 7.69 ± 0.04 (n = 3) for this experiment. This result strongly suggests the advantage of the unified capillary in accommodating a large sample volume. To check the effect of the sample matrix, the rFab was spiked at 50 nM in 50% serum and applied to the unified capillary at 0.8 psi for 4 min (Fig. 4c). Surprisingly, an almost identical electropherogram to that of the rFab in PBS-Tw was obtained, and the pI was calculated to be 7.66 ± 0.01 (n = 3). This shows that the analysis using the unified capillary is quite stable even for a sample containing serum that often impairs CIEF analysis.

A favorable feature of this coupled method is that the captured target proteins do not escape from the capillary as they are ampholytes flanked with the anode solution, an acid solution, and the cathode solution, a base solution. This means the captured protein is inevitably focused in the pH gradient without any loss. Only the protein that remains on the immunoaffinity column will escape the focusing. Generally, the first choice for elution of proteins captured on an immunoaffinity column is lowering the mobile phase pH to 2.5–3.0^20^. The anode solution, 100 mM phosphoric acid (pH 1.6), is mostly expected to elute captured proteins exhaustively. Even 10 mM phosphoric acid (pH 2.2) eluted the bound E-tagged Fab from the immunoaffinity column in an almost identical manner to 100 mM phosphoric acid, and successive irrigation with 100 mM phosphoric acid did not result in further elution. Hence all protein would appear to be eluted from the immunoaffinity column.

**Figure 5:**
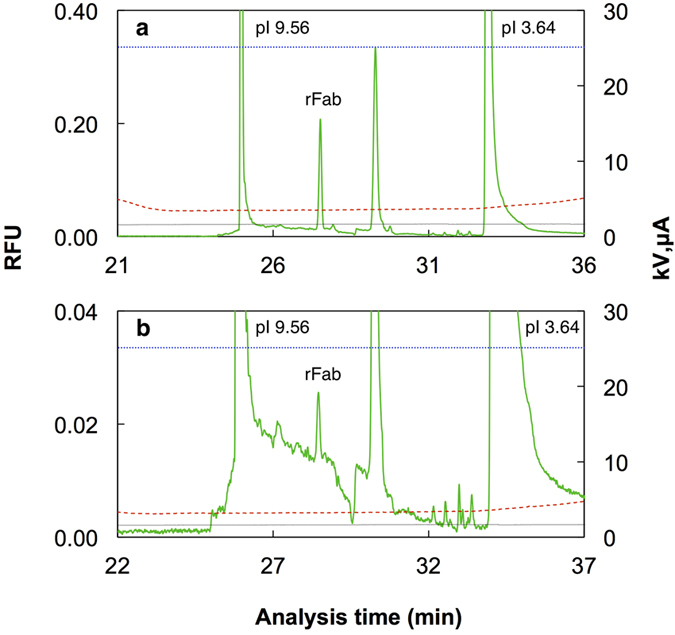
Analysis of rFab at low concentration using the unified capillary. Separation results of AIC-CIEF for the rFab in 50% serum as described in Fig. 3. Only a part of the CIEF analysis is shown. The concentration of r Fab in 50% serum was 400 pM (**a**) and 16 pM (**b**). Green solid lines, fluorescence; blue dotted lines, voltage; red broken lines, current; black lines, pressure at 0.2 psi.

**Figure 6:**
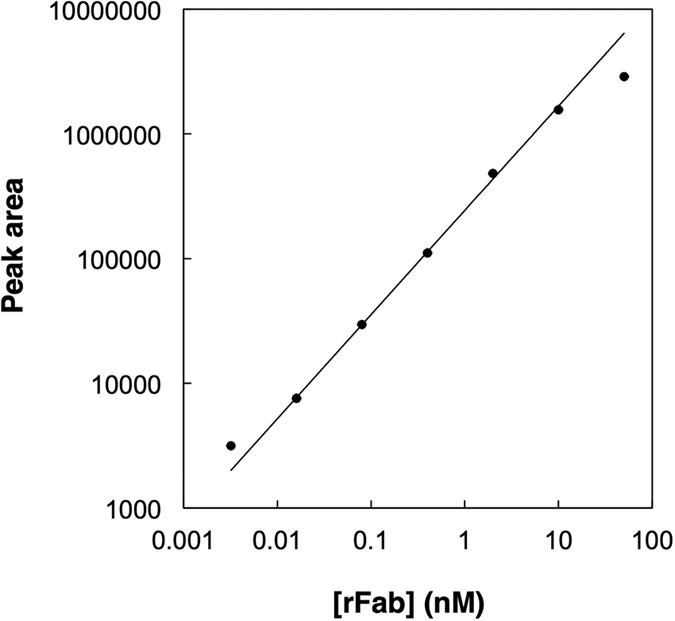
Calibration plot for the peak area against the concentration of rFab in 50% serum. Samples were injected at 0.8 psi for 4 min (0.47 μL). The values represent a single experiment (n = 1) for each concentration. The area is represented in “area unit” that is proportional to the RFU-time integral. Other conditions were the same as those described in Fig. 3.

A dilution series of rFab in 50% serum was analyzed using the unified capillary. A five-fold dilution series was made in 50% serum from 50 nM to 3.2 pM, and was subjected to IAC-CIEF analysis in the order from low to high concentration samples. As examples, the electropherograms for 400 pM and 16 pM are presented in Fig. 5. The peaks of the pI markers that were included in the carrier ampholyte solution at 2.5 nM became larger in comparison with those of rFab. Another peak detected about 2 min after the rFab peak also became more evident. This peak seemed to be an impurity originated from the pI markers, since the size of the peak in comparison with those of the pI markers did not change in the experiments with the rFab at different concentrations. During the sequential runs of analysis, the peaks of the pI markers and the rFab were detected earlier and earlier with each run. It is probable that accumulation of serum components in the unified capillary affected the EOF in the capillary. The shift in analysis time, however, is not a big issue in the identification of peaks, since peaks can be accurately identified by the calculated pI values based on the analysis time of pI markers^19^,^21^. The log-log plot for the peak area of rFab against its concentration demonstrated good linearity over almost three orders of magnitude (Fig. 6). The low value for the highest concentration sample (50 nM, 24 fmol) may be ascribed to the decrease in the capacity of the immunoaffinity column after multiple runs of analysis, 33 runs for this particular case. In a separate experiment to check the stability of the immunoaffinity column, the column was filled with 100 mM phosphoric acid and left for 16 h. During that period, the capacity decreased from 125 fmol to 22 fmol. After 33 runs, the accumulated time that the acid had been in the column was 16.5 h. Thus, it is possible that the 24 fmol of rFab sample applied in the experiment might have exceeded the reduced capacity of the immunoaffinity column. Except for this issue of the gradual decrease of capacity, the results demonstrate the high potency of IAC-CIEF in a quantitative analysis.

In repeated use of the unified capillary, the capacity of the IAC column decreased gradually. This might be due to the denaturation of the immobilized antibody caused by the immersion in the anode solution, 100 mM phosphoric acid for about 30 min in each analysis. The use of a more dilute anode solution is advantageous in maintaining the binding activity of the IAC column. On the other hand, a dilute anode solution with a low conductivity is less effective in EOF suppression during focusing and makes it more difficult to find a balanced anodic pressure with EOF using the present electrophoresis instrument that allows pressure settings with 0.1 psi difference. The use of 10 mM phosphoric acid for the anode solution is currently under investigation and will be reported in the near future.

## Conclusion

The direct coupling of IAC and CIEF is a new form of immunoelectrophoresis. It can replace a part of analysis currently performed by western blotting, with a shorter analysis time and a fully automated process.

### Further Work

To keep the use of highly sensitive laser-induced fluorescence detection, the detection of a target protein as a complex with an affinity probe, a fluorescence-labeled antibody fragment, could be an option, *i.e.*, affinity probe capillary electrophoresis^16^,^22^. The complex of the affinity probe and the target is trapped on the IAC column, separated with CIEF, and detected as the complex. The issue here is how elution with the anode solution would affect the stability of the complex. Fortunately, at least in the first case we are trying now, the complex can be successfully detected in the pH gradient. Without the use of the affinity probe, detection with UV absorption or UV fluorescence is required for the direct detection of the target. For these less sensitive detection schemes, the binding capacity of the IAC column needs to be increased. The use of disposable antibody-coated magnetic beads as an immunosorbent seems to be a promising way of increasing the binding capacity and also ruling out the issue of reduction of the capacity in the repeated use of the immunoaffinity column.

## Additional Information

**How to cite this article**: Shimura, K. and Nagai, T. Capillary isoelectric focusing after sample enrichment with immunoaffinity chromatography in a single capillary. *Sci. Rep.* **6**, 39221; doi: 10.1038/srep39221 (2016).

**Publisher's note:** Springer Nature remains neutral with regard to jurisdictional claims in published maps and institutional affiliations.
